# Artificial Intelligence for Personalized Medicine in Thyroid Cancer: Current Status and Future Perspectives

**DOI:** 10.3389/fonc.2020.604051

**Published:** 2021-02-09

**Authors:** Ling-Rui Li, Bo Du, Han-Qing Liu, Chuang Chen

**Affiliations:** ^1^ Department of Breast and Thyroid Surgery, Renmin Hospital of Wuhan University, Wuhan, China; ^2^ School of Computer Science, Wuhan University, Wuhan, China; ^3^ Institute of Artificial Intelligence, Wuhan University, Wuhan, China

**Keywords:** artificial intelligence, thyroid cancer, biomarker, personalized medicine, histopathology, fine-needle aspiration biopsy, ultrasound

## Abstract

Thyroid cancers (TC) have increasingly been detected following advances in diagnostic methods. Risk stratification guided by refined information becomes a crucial step toward the goal of personalized medicine. The diagnosis of TC mainly relies on imaging analysis, but visual examination may not reveal much information and not enable comprehensive analysis. Artificial intelligence (AI) is a technology used to extract and quantify key image information by simulating complex human functions. This latent, precise information contributes to stratify TC on the distinct risk and drives tailored management to transit from the surface (population-based) to a point (individual-based). In this review, we started with several challenges regarding personalized care in TC, for example, inconsistent rating ability of ultrasound physicians, uncertainty in cytopathological diagnosis, difficulty in discriminating follicular neoplasms, and inaccurate prognostication. We then analyzed and summarized the advances of AI to extract and analyze morphological, textural, and molecular features to reveal the ground truth of TC. Consequently, their combination with AI technology will make individual medical strategies possible.

## Introduction

Thyroid cancers (TC) have emerged in popularity over the past decades, with indolent TC accounting for the majority (1–3). For advanced TC (1, 2) and aggressive papillary thyroid carcinomas (PTC) (4), the incidence and mortality rates are also steadily increasing, which makes it imperative to adopt more effective strategies for managing such changes. In the era of personalized medicine, precise and efficient risk stratification is important before, during, and after treatment, to choose and adjust its type and intensity. The foremost step is to discover key information that reveals the biological behavior of TC. There are abundant anatomical structures (texture, internal architecture, and spatial distribution) and molecular components (gene variation, protein expression, etc.) within TC. So far, TC’s diagnosis mainly relies on image analysis (e.g., ultrasound images, cell smears, and tissue sections), but information obtained only by our naked eyes hardly enables a comprehensive analysis of the tumors (5). Given patients and their disease features, primary human cell cultures both from surgical biopsies and from fine-needle aspiration (FNA) samples foster the targeted therapies (6). However, many tough challenges still hinder a clear break of personalized treatment such as inconsistent rating ability of ultrasound (US) physicians (7), uncertainty in cytopathological diagnosis (8), difficulty in discriminating follicular neoplasms (9, 10), and inaccurate prognostication.

**Figure 1:**
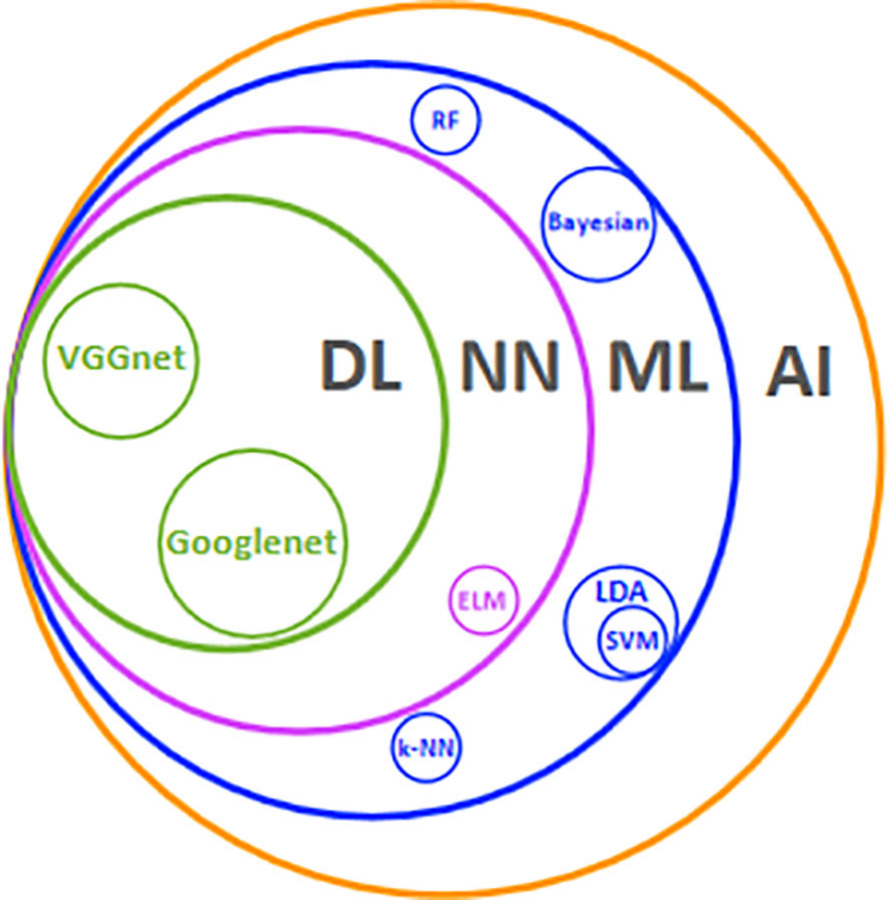
Main AI technologies and their relationships. AI. Artificial intelligence; ML, machine learning; NN, neural network; DL, deep learning; LDA, linear discriminant analysis; ELM, extreme learning machine; RF, random forest; SVM, support vector machine; k-NN, k-nearest neighbor.

Artificial intelligence (AI) is a series of technologies combined to mimic human interaction (**Figure 1**). In some tasks, it matches or exceeds human perception (11, 12). AI deals with various sorts of omics information in parallel, easily identifying and modeling a complicated nonlinear relationship in the image (13, 14). Several studies have demonstrated that AI classifier is comparable to radiologists while qualitatively analyzing thyroid nodules (TN) (15–18). Furthermore, AI can extract and quantify key image information, whereby image diagnosis converts from a subjective qualitative task to objective quantitative analysis. This more detailed and precise information is conducive to special risk stratification and propels tailored management to transit from the surface (population-based) to a point (individual-based).

**Figure 2:**
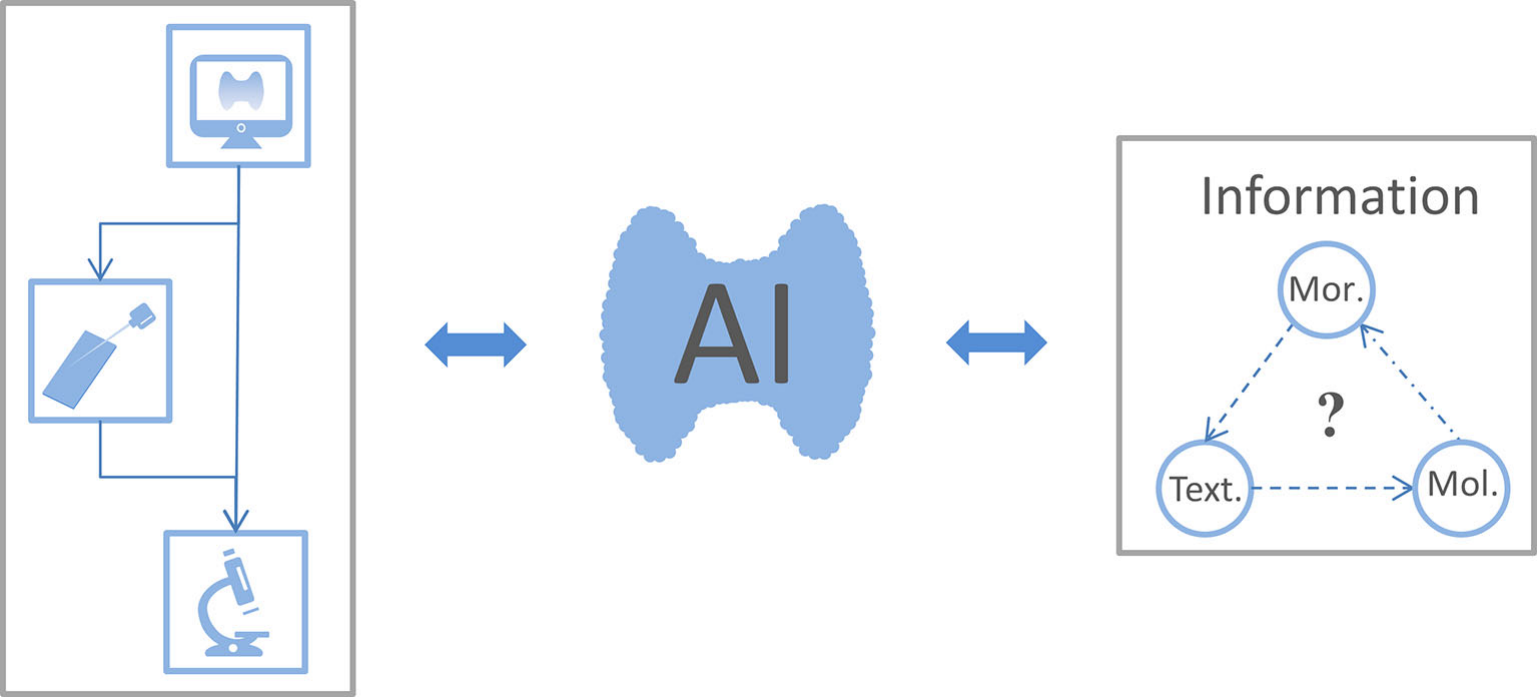
The connection between the focus reviewed. Thyroid ultrasound is the preferred imaging examination for patients with thyroid nodules. When sonographers consider certain thyroid nodules as malignant, the patient could choose fine-needle aspiration biopsy or surgery for a confirmed diagnosis. Artificial intelligence (AI) uses ultrasound images, cell smears, and tissue slices to extract morphological, textural, and molecular features. This information is fed back into the AI classifier to improve its performance and thus optimize thyroid cancer diagnosis and treatment workflow. As expected, whether these morphological (Mor.), textural (Text.), and molecular (Mol.) features are related to each other warrants further study.

In this review, we aimed to summarize the use of AI for extracting and analyzing morphological, textural, and molecular features to reveal detailed information and personalize therapies for TC patients (**Figure 2**).

## Applications of AI in the US Diagnosis of TN

TN with several typical ultrasound features implies an increased risk of malignancy, such as solid composition, hypoechogenicity, irregular margin, microcalcification, and taller-than-wide shape. However, these properties can neither confirm nor exclude the diagnosis of TC (19). The observer’s agreement among multiple centers is poorly satisfactory in assessing these features (7). Thyroid Imaging Reporting and Data Systems (TI-RADS) are enormously valuable to PTC as risk stratification systems, while relatively less to FTC, MTC, and other malignancies (20). Interestingly, the AI model appears to be a promising tool to facilitate a better knowledge of TN via quantitative analysis of typical US features and introduction of texture features.

### Performance of Typical US Features

Wildman-Tobriner et al. (21) developed an AI TI-RADS based on the American College of Radiology (ARC) TI-RADS. This system optimized the evaluation task through reassigned values for eight ultrasound features, highlighting the status of hypoechogenicity or marked hypoechogenicity. The novel AI TI-RADS had better accuracy than ARC TI-RADS when performed by inexperienced radiologists (55% vs. 48%) and experts (65% vs. 47%). Similar to other studies, ARC TI-RADS-based classifiers had higher sensitivity and slightly lower specificity (21–24). Wu et al. (25) evaluated quantitative echoic indexes for detecting malignant TN, which showed higher accuracy than typical ultrasound hypoechogenicity (>60% vs. 54.01%). We summarized the outcomes of the ultrasound features employed by AI for classification in **Table 1** and found the most widely used features were shape, margin, echogenicity, calcification, composition, and size. In other words, these discriminative features seem to be the focus for the AI model to learn (31, 37). Particularly, Choi et al. (30) demonstrated several new calcification features associated with TN malignancy, including shorter calcification distance ratio, smaller amounts of calcification, and dimmer calcification. Chen et al. (28) quantified TN malignant risk through the calcification index. These new features boosted diagnostic accuracy by combining qualitative and quantitative methods (30, 38). Current AI classifiers focus on benign and malignant TN dichotomy, and certain of them like the S-Detect series have already become commercially available (32, 34). Furthermore, they are expected to predict more tumor-biological behaviors such as lymph node metastasis (39, 40) and pathological subtypes (41).

**Table 1:** Summary of key studies on the outcome of ultrasound features in artificial intelligence classifier identifying benign and malignant thyroid nodules.

Study	Patients	Features	Classifiers	Accuracy, %	Sensitivity, %	Specificity, %	AUC
Lim et al. (26)	96	Size, margin, cystic change, echogenicity, and macrocalcification	ANN	93.78	NA	NA	0.949
Savelonas et al. (27)	387	Boundary features	SVM	NA	NA	NA	0.95
Chen et al. (28)	256	Calcification index	AmCAD-UT	NA	NA	NA	0.746
Zhu et al. (29)	618	Not well-circumscribed, solid, hypoechogenicity, microcalcification, taller than wide, absent peripheral halo	ANN	83.10	83.80	81.80	0.828
Choi et al. (30)	85	Quantitative calcification	NN	82.80	83.00	82.40	0.83
Wu et al. (25)	333	Quantitative echogenetic values	AmCAD-UT	70.32	33.12	93.31	NA
Xia et al. (31)	187	Margin, shape, composition, echogenicity, and calcification	ELM	87.72	78.89	94.55	0.867
Choi et al. (32)	89	Size, margins, shape, composition, echogenicity, orientation, and spongiform	S-Detect 1 (SVM)	81.40	90.70	74.60	0.83
Ouyang et al. (33)	1036	Size, margins, shape, composition, echogenicity, calcification, aspect ratio, capsule, hypoechoic halo, vascularity, and cervical lymph node status	RF + k-SVM	NA	NA	NA	0.954
Kim et al. (34)	106	Size, margins, shape, composition, echogenicity, calcification, orientation, and spongiform	S-Detect 2 (CNN)	73.40	81.40	68.20	NA
Liu et al. (35)	4655	Shape, context, and margin	CNN	94.90	97.20	89.10	NA
Wildman-Tobrineret et al. (21)	1264	ACR TIRADS	Genetic Algorithm	65.00	93.30	64.70	0.93
Guan et al. (36)^a^	2235	Margin size	Inception-v3	90.50	93.30	87.40	0.956
Zhao et al. (22)	822	Size + ACR TIRADS	ML	82.10	90.90	78.10	0.917
Jin et al. (23)	695	ACR TIRADS	CNN	80.35	80.64	80.13	0.87
Bai et al. (24)	13984^b^	ACR TIRADS	CNN	88.00	98.10	79.10	NA

ANN, artificial neural network; SVM, support vector machine; AmCAD-UT, a software integrating AI technology and clinicians’ expertise; NN, neural network; ELM, extreme learning machine; RF, random forest; k-SVM, kernel support vector machine; Inception-v3, a kind of Googlenet; CNN, convolutional neural network; ML, machine learning; NA, not available; ACR TRIADS, American College of Radiology Thyroid Imaging Reporting and Data System, the features included in this system is composition, echogenicity, shape, margin, and echogenic foci.

a This study focused on the classification between papillary thyroid carcinomas and benign nodules.

b Nodules, not patients.

### Performance of Texture Features

A meta-analysis suggested that a taller-than-wide shape displays TN’s variation in space and orientation growth, and it is defined as the most suggestive feature for malignancy (42). Texture features refer to the characterization of spatial distribution and surface orientation with numerical features (43). Thus, texture analysis as a powerful alternative will make it possible for radiologists to comprehend the TN in depth and gain a correct diagnosis. Raghavendra et al. (44) integrated spatial and fractal texture features and screened two features with an excellent area under the curve in diagnostic practice (94.45%). Prochazka et al. (45) used AI to extract texture features from US images independent of the direction of the US probe and achieved better accuracy (94.64%). Yu et al. (46) performed a numerical transformation of two US features, unregulated shape and long/short-axis ratio into the perimeter2/area and the angle between the long axis and the horizontal axis. These new features showed excellent sensitivity and specificity (100% and 87.88%, respectively) combined with 65 texture features. Collectively, AI mode has a role in integrating typical ultrasonic and texture features, and this fusion might sharply reduce the differences in judgments among US professionals. Despite the mounting advantages of the AI model in optimizing and even creating workflows, many remarkable factors hold its ultimate practice back in the real world. The three main factors are as follows: (i) poor availability of large-high-quality datasets to guarantee great robustness (17); (ii) lack of explainability for conclusions from a black-box algorithm to solidify the trust between physicians and patients (47, 48); (iii) financial burden from specific equipment and research costs (48).

## Applications of AI in Cytopathological Evaluation From FNA

FNA is a primary preoperative examination to evaluate TN. Its report system, the Bethesda System for Reporting Thyroid Cytopathology (TBSRTC), is a state-of-the-art and category-based method for clinicians’ decision-making. While TBSRTC includes six diagnostic categories on the estimated risk of malignancy (ROM) (**Table 2**), 15%–30% of TN continues to be classified as indeterminate TN (ITN), most frequently TBSRTC categories III, IV, and V (8). Recent studies showed excellent consistency between machine learning (ML) models and cytologists in malignancy prediction (49–51), in which the ROM of TBSRTC III determined by the ML model was considerably lower than by manual classification (4.2% vs. 18.8%) (51). It’s worth noting that morphological and genetic classifications assisted by the AI model are fairly accurate at distinguishing malignancy from benign TN (52–54) (**Table 3**).

**Table 2:** The 2017 TBSRTC categories and their own risk of malignancy.

Diagnostic category	Risk of malignancy if NIFTP≠ CA (%)
**I. nondiagnostic or unsatisfactory** Cyst fluid only Virtually acellular specimen Other (obscuring blood, clotting artifact, etc.)	5–10
**II. Benign** Consistent with a benign follicular nodule (includes adenomatoid nodule, colloid nodule, etc.) Consistent with lymphocytic (Hashimoto) thyroiditis in the proper clinical context Consistent with granulomatous (subacute) thyroiditis Other	0–3
**III. atypia of undetermined significance or follicular lesion of undetermined significance**	6–18
**III. follicular neoplasm or suspicious for a follicular neoplasm** Specify if Hürthle cell (oncocytic) type	10–40
**IV. suspicious for malignancy** Suspicious for papillary carcinoma Suspicious for medullary carcinoma Suspicious for metastatic carcinoma Suspicious for lymphoma Other	45–60
**V. Malignant** Papillary thyroid carcinoma Poorly differentiated carcinoma Medullary thyroid carcinoma Undifferentiated (anaplastic) carcinoma Squamous-cell carcinoma Carcinoma with mixed features (specify) Metastatic carcinoma Non-Hodgkin lymphoma Other	94–96

This is an integrated table from reference (8).

**Table 3:** The main performance of artificial intelligence using pathological information in different task.

Study	Subject	Test	Feature	Task	Classifier	Accuracy,%
Cochand-Priollet et al. (54)	157	FNA	Nuclear size, shape, and texture	Classification of malignant and benign TN	FNN	89.00
Daskalakis et al. (53)	115	FNA	Nuclear morphology and texture	Classification of malignant and benign TN	k-NN + PNN + Bayesian	95.70
Tomei et al. (52)	93	FNA	mRNA expression	Classification of malignant and benign TN	BNN	88.80
Sanyal et al. (55)	544	FNA	Nuclear morphology and papillary structure	Classification of PTC and non-PTC	ANN	85.06
Guan et al. (56)	279	FNA	Nuclear contour	Classification of PTC and benign TN	VGG-16	97.66
Savala et al. (57)	57	FNA	Cellular and nuclear morphology	Classification of FC and FA	ANN	100.00
Alexander et al. (58)	249^a^	FNA	RNA expression	Classification of malignant and benign ITN	SVM	65.00
Patel et al. (59)	183^a^	FNA	RNA sequencing	Classification of malignant and benign ITN	SVM	74.00
Nikiforova et al. (60)	175^a^ ^b^	FNA	Genetic alterations	Classification of malignant and benign ITN	Torrent Suite software	90.90
Lithwick-Yanai et al. (61)	150^a^ ^b^	FNA	MicroRNA expression	Classification of malignant and benign ITN	LDA + k-NN	83.65
Sun et al. (62)	64^a^	FNA	Protein	Classification of malignant and benign TN	ANN	87.53
Wang et al. (63)	10	Histo.	Nuclear size and chromatin concentration	Classification of FC, FA, and normal thyroid	SVM	100.00
Ozolek et al. (64)	94	Histo.	Nuclear morphology	Classification of five follicular lesions	LDA +k-NN	100.00^c^
Zhao et al. (65)	800	Histo.	Gene variant pathways	TC risk stratification	ANN	77.50/86.00^d^
Ruiz et al. (66)	495	Histo.	Gene signature	Prediction of lymph-node metastasis and disease-free survival	LDA	82.63

FNA, fine-needle aspiration biopsy; Histo., Histopathology; TN, thyroid nodules; FNN, feedforward neural network; PNN, probabilistic neural network; BNN, Bayesian neural network; PTC, papillary thyroid carcinoma; FC, follicular carcinoma; FA, follicular adenoma; ANN, artificial neural network; SVM, support vector machine; LDA, linear discriminant analysis; k-NN, k-nearest neighbor; NA, not available.

a Only the validation cohort is included, which in the study by Lithwick-Yanai et al. was specifically the set agreed upon by the three pathologists.

b FNA smears, not patients.

c The accuracy in the group of FA vs. FC, FA vs. NG, FC vs. NG, FA vs. FV-PTC, and FC vs. WIFC.

d The accuracy of recognizing the different- risk cases was 77.50% (low-risk) and 86.00% (high-risk) respectively.

### Performance of Morphological Features

PTC, the most common TC (>80%), arises from abnormal growth of thyroid epithelial cells (28, 38). In recent years, AI models with quantitative morphological features have tried to improve follicular lesions’ recognition capacity (55–57). Sanyal et al. (55) obtained the nuclear morphology and papillary structure of PTC under two magnifications (×10 and ×40). CNN model selected PTC from colloid goiter, follicular neoplasms, and lymphocytic thyroiditis by right of these features. Guan et al. (56) developed a new AI cytological classification based on nuclear size and staining information (the contours, perimeter, area, and means of pixel intensity), whose results showed high accuracy (97.66%) to differentiate PTC from benign nodules. Another research group also confirmed this performance (57). They first derived nuclear pleomorphism and area information and then reported the weight of 17 cytological and morphological features. Finally, their model successfully discriminated follicular carcinoma (FC) from follicular adenoma (FA) (57) (**Table 3**).

The major difference between FC and FA is the occurrence of capsular or vascular invasion (67). Preoperative examinations of both US and FNA have difficulty in making a reliable diagnosis. A highly vascularized tumor protrusion on the US strongly indicates FC, which is rather rare yet (68). Seo et al. (69) took full advantage of this difference by collecting information about the tumor edge in the US images. The overall accuracy was 89.51% for distinguishing FC and FA. Yang et al. (70) segmented the whole lesions of follicular neoplasms; as a result, the classification accuracy was significantly improved to 96%. This clarified the importance of internal information and affirmed the study’s reliability by Savala et al. (57). Similarly, the diagnosis of MTC and ATC is histology dependent (71, 72), yet now no studies to our knowledge have answered the hope of AI in their ultrasound and cytopathological diagnosis.

### Performance of Biomolecules

For patients with ITN, repeat FNA or lobectomy might be performed because management guidelines are more flexible (8, 73). Fortunately, molecular tests provide a noninvasive and accurate option to reduce clinical and healthy uncertainty (8, 67). Each genome contains as much information as 100,000 photographs (74). Next-generation sequencing (NGS) can perform high-speed analysis of multiple genes parallelly in a single operation, producing billions of molecular fragments (74, 75). It has always been a crucial component of big data due to its large volume of data, the astonishing velocity of the sequencing methods, and the result output’s veracity. Traditional information systems are less competent to analyze large and complex datasets (76, 77). AI as a big data algorithm can integrate multi-omic data in a different learning task, and automatically realize high-level features’ detection or classification (77). Some genetic classifiers have played their strengths in TN such as the Afirma gene expression classifier (GEC) (58), gene sequence classifier (GSC) (59), gene mutation-based classifier (ThyroSeq) (60, 78), and microRNA-based classifier (RosettaGX Reveal) (61, 79). The GEC involved 167 genes that displayed high sensitivity (92%) and positive predictive value (PPV: 93%) but limited by its relatively low specificity (52%) and negative predictive value (NPV: 47%) (58). GSC expanded the gene spectrum to 10,196 genes by RNA-enhanced NGS. Compared to GEC in the same samples, it made progress in screening for benign nodules (sensitivity: 91.1% and specificity: 68.3%) (59) (**Table 3**). These two classifiers are the most broadly accepted methods to rule out malignant nodules. In general, ThyroSeq and RosettaGX Reveal are more like rule-in entities. Nikiforova et al. (60) achieved a robust sensitivity of 98% and a hopeful specificity of 81.8% by employing the latest version of ThyroSeq (ThyroSeq V3) to recognize a few cancers from most benign tumors. Steward et al. (78) drew similar conclusions in a prospective blinded multicenter study and reported 94% sensitivity and 82% specificity. RosettaGX Reveal showed 98% sensitivity and 78% specificity when validated in independent cases with all three pathologists’ agreement on the histopathological diagnosis (61) (**Table 3**). However, whether the mentioned classifiers could consolidate and complement each other remains so ambiguous that we need to further investigate the precise application strategy.

The multi-gene analysis is able to enhance diagnostic performance, but it may be limited due to key genes’ deletion or their reduced expression. Of note, the number of thyroglobulins has been considered as a predictor of postoperative disease progression (67). Therefore, the key proteins might provide some added information for personalized therapy. Recent research has confirmed that proteins are more stable than RNA in clinical tissues (80). Sun et al. (62) completed a 14 protein-based ANN classifier for TN classification. This model realized the accuracy of 90.62% and 87.53% in multicenter retrospective and prospective samples respectively (**Table 3**). Some molecular alterations such as *BRAF* mutations (81) are diagnostic of cancer, but most of the other alterations (82, 83) show overlap in both benign and malignant lesions. Therefore, assessing the risk of malignancy by molecular testing should depend on knowledge of the prior cytological appearance.

## Applications of AI in Histopathological Analysis

Upon reliable evidence obtained by the US and FNA examination, tumor information from the resected specimens is significant for pathologists to diagnosis TC such as tumor size, pathologic types, and degree of malignancy. Molecular patterns in the tumor microenvironment like cytokines, chemokines, and adipocytokines interconnect the units of immune-inflammatory responses (e.g., macrophages, neutrophils, lymphocytes) and tumor nest (e.g., epithelial cancer cells, fibroblasts, endothelial cells) (84). The more detailed information the pathologists provide, the more precise the treatment strategies physicians take. The combination of AI, morphology, and molecular markers is expected to provide more information for TC management at a patient’s level.

### Performance of Morphological Features

The morphological feature is the final station of biological behavior and genetic variation of TN. The morphological performance supported by AI might be beneficial for the accurate diagnosis of TN. Wang et al. (63) successfully classified FA, FC, and normal tissues according to nuclear size and chromatin concentration. Ozolek et al. (64) achieved nearly perfect accuracy based on nine nuclear morphological features for discriminating five thyroid follicular lesions: FA, FC, follicular variant of PTC, nodular goiter, and the widely invasive FC (**Table 3**). However, further validation of these models is required due to tumor complicated heterogeneity, which was also turned out in a recent study for classifying TC, normal tissues, nodular goiter, and adenomas using a deep learning model (85).

Morphologically, FV-PTC is a mixed entity for typical PTC nuclear features and entirely or almost entirely follicular growth patterns. FV-PTC includes two major subtypes: encapsulated (EFV-PTC) and non-encapsulated or infiltrative variants (IFV-PTC) (86). The former generally have RAS mutations like follicular tumors, the latter often presents extrathyroidal extension (ETE), lymphatic metastasis, and BRAF mutations like classical PTC (cPTC) (87). Likewise, EFV-PTC usually appears invasive or non-invasive, and the noninvasive encapsulate tumor was redefined from carcinoma to borderline tumor, noninvasive follicular thyroid neoplasm with papillary-like nuclear features (NIFTP) (86). Up to a point, the invasive EFV-PTC behaves more aggressively like FC, whereas NIFTP is with indolent clinical behaviors like FA (87). It is believed that invasive EFV-PTC might develop from NIFTP (88). Borrelli et al. (89) revealed a significant difference in miRNA expression of FA, NIFTP, and IFV-PTC. In particular, just two miRNA (miR-10a-5p and miR-320e) enable us to differentiate NIFTP from IFV-PTC. In another study by Selvaggi (90), none of the multinucleated giant cells (MGCs) were observed in 20 NIFTP cases, while the amount of MGCs varied from 1 to 4 in 88% of the FVPTC cases (both IFV-PTC and invasive EFV-PTC). When utilizing computer quantitative analysis to classify FV-PTC, Chain et al. (91) demonstrated the NIFTP nuclear area (mean, 54.8 μm2) and elongation was smaller than PTC (mean, 77.2 μm2); Hsieh et al. (92) addressed PD-L1 expression in NIFTP was lower than in invasive EFV-PTC. These quantitative morphological characteristics and definite molecular alterations contribute to FV-PTC classification.

As FV-PTC’s definition stated, the coexistence of papillary and variable follicular structures is so common in cancer nests that we hold a positive view about more transitional or intermediate categories between the cPTC and FV-PTC. Undoubtedly, the clearer the learning exemplars, the easier it is to learn for the AI model because it receives fewer error messages (13). For greater efficiency, it’s essential to accurately classify the training set and refine the output target.

### Performance of Genetic Parameters

The American Thyroid Association risk stratification system and the American Joint Committee on Cancer TNM staging system are used to guide postoperative treatment and predict post-treatment outcomes, which incorporate several parameters including age, ETE, anatomic location, number, and size of metastatic lymph nodes, aggressive variants, vascular invasion, and distant metastasis. Nonetheless, these systems fail to routinely recommend a genetic determination to guide individual management (67, 93). Zhao et al. (65) selected 10 gene variant pathways that involved inflammatory and immune responses to determine the TC patients’ risk level. Based on these pathways, the patients were divided into the high-risk and low-risk groups whose survival time was significantly better than the former. Ruiz et al. (66) demonstrated a 25-gene panel related to molecular pathways, cell structure, and function was an independent prognostic factor for lymphatic metastasis and disease-free survival (**Table 3**). Further evidence is still warranted to address the value of this genetic information to TN’s triage and biological behaviors. As AI and gene testing technology upgrade, the cooperation of traditional clinic-pathological parameters and gene molecules might yield more precise therapeutic implications.

## Conclusion

The future development of personalized medicine in TC still faces several challenges like inconsistent rating ability of US physicians, uncertainty in cytopathological diagnosis, difficulty in discriminating follicular lesions, and inaccurate prognostication. AI’s application has improved the efficiency and accuracy of diagnosis and treatment in other tumors (94–96). A growing amount of medical information can be extracted and analyzed through AI technology. This review has innovatively offered ideas for the ultrasonic and pathological testing out of these dilemmas in terms of morphological, textural, and molecular features. As more key parameters are explored from the tumor and its microenvironment, the AI-aided combination of morphological and molecular features will pave the way for TC’s protocol at the individual level.

## Author Contributions

L-RL: study design, literature review, article writing, and revision. BD: literature review. H-QL: literature review. CC: study design, article revision. All authors contributed to the article and approved the submitted version.

## Funding

This research was supported by the grants from the Fundamental Research Funds for the Central Universities (2042019kf0229) and the Science and Technology Major Project of Hubei Province (Next-Generation AI Technologies) (2019AEA170).

## Conflict of Interest

The authors declare that the research was conducted in the absence of any commercial or financial relationships that could be construed as a potential conflict of interest.
